# Comment on “Mortality Related to Different Circulatory Diseases Before, During and After the COVID‐19 Pandemic”

**DOI:** 10.1002/clc.70449

**Published:** 2026-08-15

**Authors:** Abhinit Kumar, Ranjana Roy, Parappa Sajjan, Alok Bhatt, Jeffrin Reneus Paul

**Affiliations:** ^1^ Department of Psychiatry, School of Medical Sciences and Research Sharda University Greater Noida India; ^2^ Dr. D. Y. Patil Medical College, Hospital and Research Centre Dr. D. Y. Patil Vidyapeeth (Deemed‐to‐be‐University), Pimpri Pune Maharashtra India; ^3^ Department of Public Health, Malla Reddy Institute of Dental Sceinces Malla Reddy Vishwavidyapeeth (Deemed to be University), Suraram Hyderabad Telangana India; ^4^ Faculty of Pharmaceutical Sciences Graphic Era Hill University Dehradun India; ^5^ Centre for Promotion of Research Graphic Era Deemed University Dehradun India; ^6^ Saveetha Medical College and Hospital, Saveetha Institute of Medical and Technical Sciences Saveetha University Chennai India

## Conflicts of Interest

The authors declare no conflicts of interest.

Dear Editor,

Capodaglio and colleagues [1] provide valuable population‐level evidence using multiple causes of death (MCOD), seasonal autoregressive integrated moving average models, and Lee‐Carter sensitivity analyses. Their conclusion that cardiovascular mortality in 2023 largely returned to pre‐pandemic trends is plausible. However, the strength of that inference depends on two linked choices: how a cardiovascular‐related death is defined and how the counterfactual trajectory is constructed.

Counting any mention of ischemic heart disease, cerebrovascular disease, hypertensive disease, or atrial fibrillation improves recognition of multimorbidity, but MCOD rates are also sensitive to death‐certification and co‐mention practices [2]. During and after COVID‐19, changes in whether chronic cardiovascular disease is selected as the underlying cause, listed as a contributing condition, or co‐recorded with COVID‐19 could alter apparent trends without an equivalent change in disease incidence. A paired analysis of underlying‐cause and MCOD rates, the annual MCOD‐to‐underlying‐cause ratio, and the proportion of certificates with COVID‐19 co‐mentioned would help distinguish epidemiological recovery from certification effects. This matters because underlying‐cause analyses have shown divergent 2023 patterns across cardiovascular subtypes [3].

The counterfactual is similarly consequential. The primary forecast uses the entire 2008‐2019 period, although the pre‐pandemic trajectories differ markedly among the four conditions and may not remain stable across alternative baseline windows. Methodological comparisons in the same regional setting have shown that excess‐mortality estimates vary with the selected baseline and modeling approach [4]. Rolling‐origin validation through 2019, forecasts based on shorter windows such as 2010−2019 and 2015−2019, and an ensemble or interrupted‐time‐series sensitivity analysis would quantify this dependence. Conversely, the 2008‐2022 model includes the pandemic shock in its fitting period; therefore, observed 2023 rates below that forecast should not be treated as an independent confirmation of recovery. The most defensible wording is that 2023 rates were consistent with, rather than proved to have returned to, the pre‐pandemic counterfactual.

Finally, the December 2023 peak is described as associated with simultaneous circulation of respiratory viruses. European surveillance documented temporal coincidence between excess mortality and COVID‐19, influenza, and respiratory syncytial virus activity [5], but the present analysis did not directly test that attribution. Incorporating viral‐surveillance indicators, temperature, and COVID‐19 co‐mention as exogenous covariates, ideally with age‐ and sex‐stratified estimates, would clarify whether the late‐2023 signal reflected infection, seasonality, or residual model error. These additions would not negate the reassuring findings; they would sharpen whether 2023 represents epidemiological normalization, certification normalization, or both.

## Data Availability

The data that support the findings of this study are available on request from the corresponding author. The data are not publicly available due to privacy or ethical restrictions.
